# Cutaneous reactions after CoronaVac and BioNTech vaccines

**DOI:** 10.1111/jocd.15573

**Published:** 2022-12-27

**Authors:** Ümran Öner, Akın Aktaş

**Affiliations:** ^1^ Department of Dermatology, School of Medicine Kastamonu University Kastamonu Turkey; ^2^ Department of Dermatology, School of Medicine Ankara Yıldırım Beyazıt University Ankara Turkey

## ETHICAL APPROVAL

Authors declare human ethics approval was not needed for this study.

Dear Editor,

The first COVID‐19 vaccine to humans worldwide was administered in the UK on December 20, 2020 (BioNTech). In our country, vaccination started on January 13, 2021 (CoronaVac). As the vaccination rate increases worldwide, cutaneous reactions such as local injection site reactions, urticaria, cutaneous eruptions, herpes simplex, herpes zoster, pernio, and reactions related to cosmetic filler injections are reported.^1^, ^2^, ^3^

We present nine patients who applied to our dermatology outpatient clinic because of skin rash within 15 days after being vaccinated with CoronaVac or BioNTech between April 2021 and July 2021 and did not have a chronic disease or drug use history other than vaccination in the last month. (Written informed consent from the patients for the use of images and publication of their details were obtained.)

**FIGURE 1:**
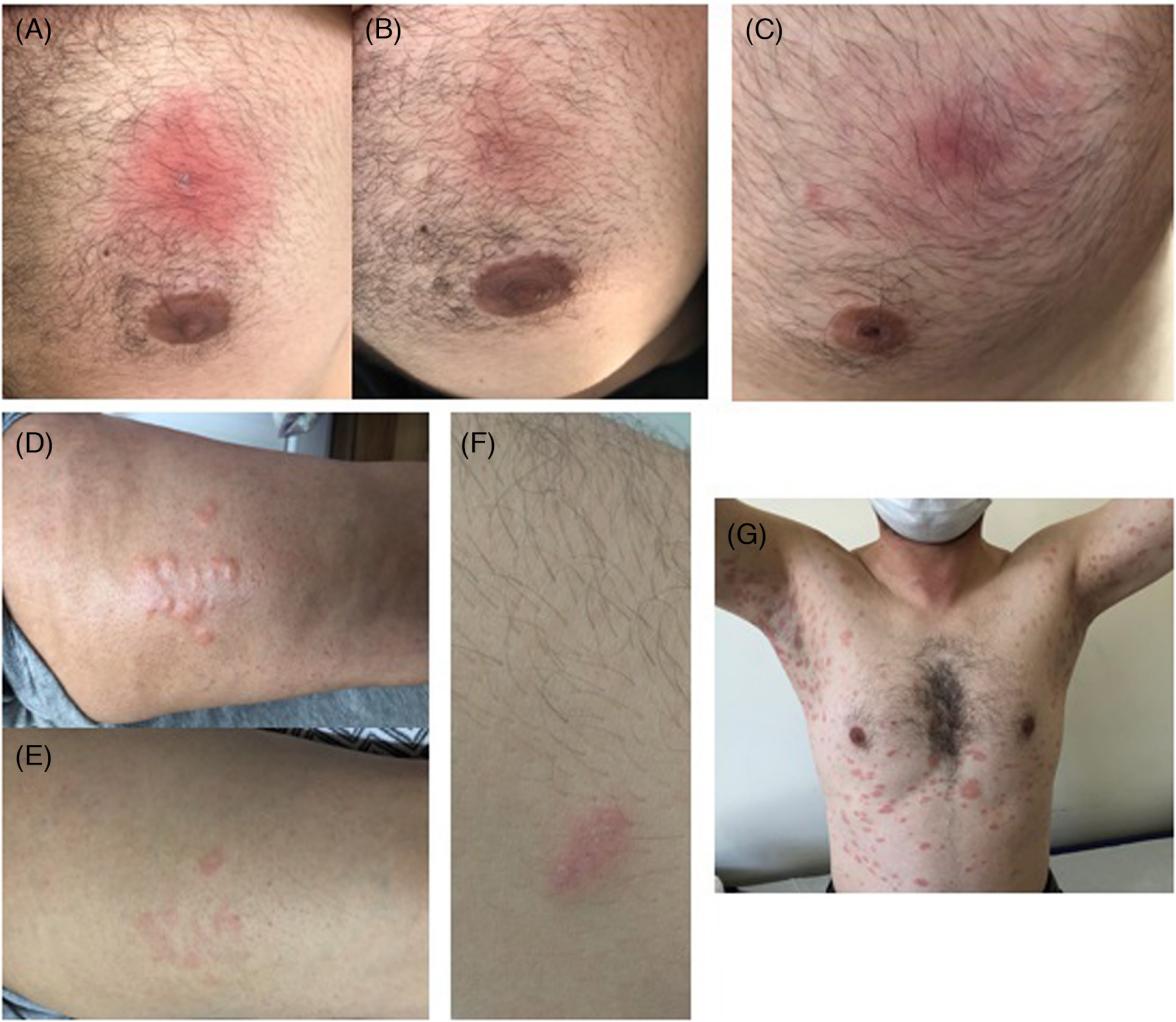
(A) Patient 1—localized erythema on the anterior trunk, (B) Patient 1—regressed lesion after one week, (C) Patient 2—localized erythema on the anterior trunk, (D) Patient 3—urticarial papules on the lower extremity, (E) Patient 3—regressed lesions after 3–6 h, (F) Patient 4—grouped vesicles on the erythematous ground, (G) Patient 5—erythematous plaques with collarette scaling on the trunk and upper extremities.

**FIGURE 2:**
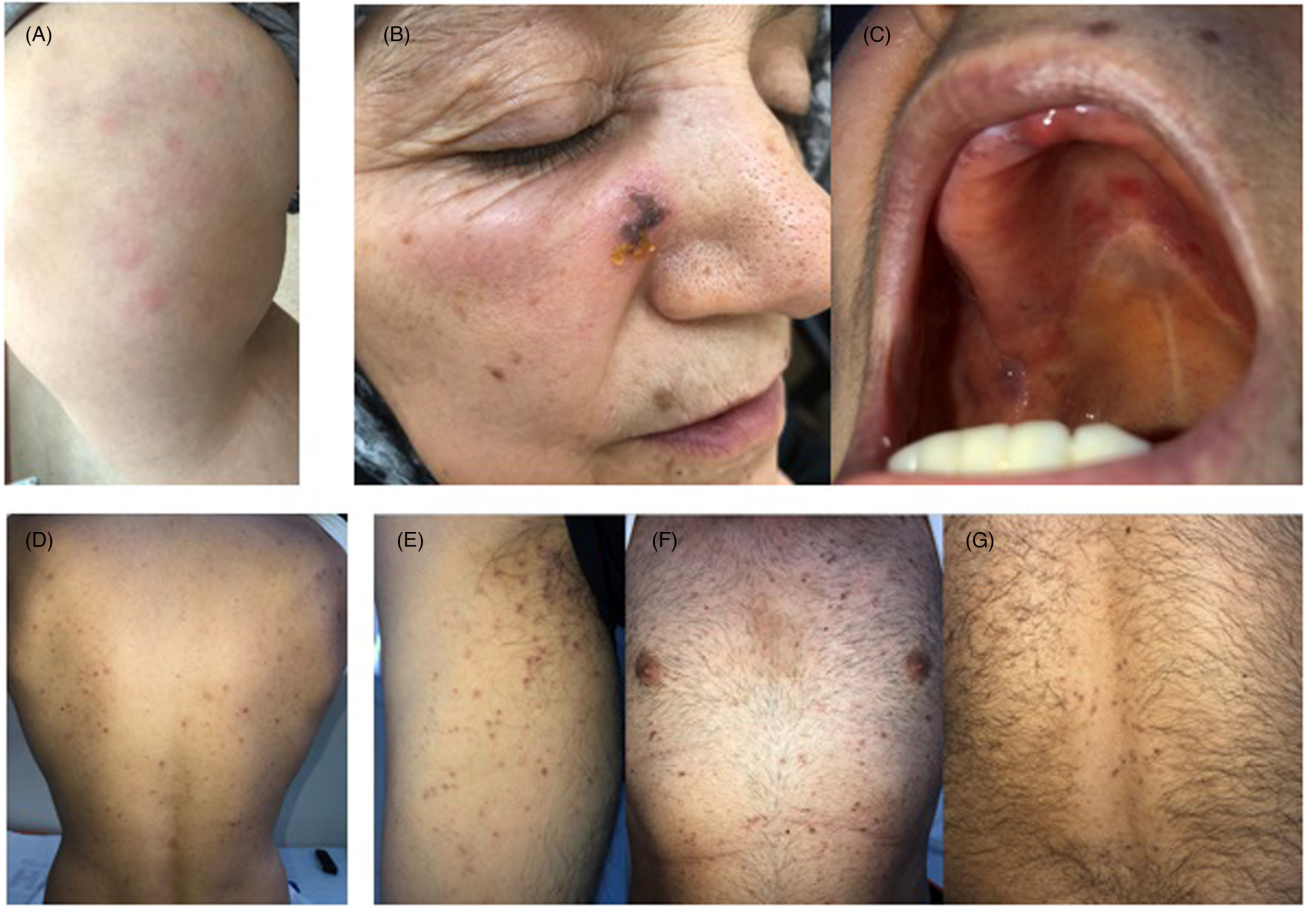
(A) Patient 6—urticarial plaques on the lower extremity (3 days after the second dose of CoronaVac), (B, C) Patient 7—painful crusted vesicles on the malar region and painful eroded lesions on the palate, (D) Patient 8—papulopustular lesions on the posterior trunk, (E–G) Patient 9—lichenoid papules on the upper extremity and trunk.

The average age of the six male and three female patients who applied with a complaint of skin rash following vaccination was 38.3 years. The mean time to appearance of the rash was four days. Five patients applied with the cutaneous reaction after the BioNTech and four after the CoronaVac (Table 1). The earliest finding was localized erythema diagnosed with fixed drug eruption on the anterior side of the trunk that started 3–4 h after the first dose of BioNTech in a 19‐year‐old male patient and gradually increased (Figure 1). The latest reaction was localized erythema on the anterior trunk, similar to the previous patient, 15 days after the first dose of BioNTech. Other cutaneous reactions after BioNTech were acute urticaria, pityriasis rosea‐like eruption, and herpes simplex (Figure 1). The cutaneous reactions after CoronaVac were acute urticaria, papulopustular eruption, lichenoid eruption, and herpes zoster (Figure 2).

**TABLE 1:** Patients with cutaneous reactions after vaccination

	Age	Gender	Cutaneous reactions	Vaccine‐dose	Time after vaccination
Patient 1	19	M	Fixed drug eruption	BioNTech‐ 1st	3–4th hour
Patient 2	28	M	Fixed drug eruption	BioNTech‐ 1st	15th day
Patient 3	37	F	Acute urticaria	BioNTech‐ 1st	1st day
Patient 4	35	M	Herpes simplex	BioNTech‐ 1^st^	2nd day
Patient 5	40	M	Pityriasis rosea‐like eruption	BioNTech‐ 1st	7th day
Patient 6	63	F	Acute urticaria	CoronaVac‐ 1st CoronaVac‐ 2nd	10th day 3rd day
Patient 7	67	F	Herpes zoster	CoronaVac‐ 3rd	2nd day
Patient 8	26	M	Papulopustular eruption	CoronaVac ‐1st	4th day
Patient 9	30	M	Lichenoid eruption	CoronaVac‐ 1st	5th day

Abbreviations: F, Female; M, male.

Cebeci et al.^4^ reported the first cutaneous reaction after CoronaVac in a female patient with petechial lesions on the lower extremities as a vaccine‐induced hypersensitivity reaction and stated that no symptoms developed after the second dose of the vaccine.

Seven hundred eighty healthcare workers from Turkey were evaluated four weeks after vaccination with CoronaVac, and only 2.3% of them developed cutaneous side effects (1.5% cutaneous eruption, 0.8% urticaria). It was stated that the most common sites of involvement were the trunk (42%) and the face (36%), and females were more frequently affected than males.^2^ Similarly, in another study evaluating the cutaneous side effects of CoronaVac, it was stated that females were affected more frequently.^5^ On the contrary, in our patients, the female: male ratio was 1:2.

A study evaluating cutaneous reactions after BioNTech and Moderna reported that the most common cutaneous side effects related to BioNTech were local injection site reactions and urticaria. The reaction occurred after the first dose in 34 individuals and after the second in 40 individuals. Among the patients who had a cutaneous reaction after the first dose and had the second dose, only 43% of them developed a cutaneous reaction again.^3^ Avallone et al. suggested that any cutaneous reactions occurring within 30 days after the SARS‐CoV‐2 booster dose were considered as potentially related to anti‐COVID‐19 vaccines.^1^ Among our cases, the most striking skin finding after the first dose of BioNTech was the fixed drug eruption, and we have not encountered a case related to COVID‐19 vaccines previously reported in the literature.

One of the cutaneous reactions reported due to COVID‐19 vaccines is herpes virus infections. Catala et al.^6^ have suggested that COVID‐19 vaccines are associated with the reactivation of herpes viruses and the possible mechanism is that a strong specific immune response against the S protein from vaccines disrupts cell‐mediated control of these latent viruses. Varicella‐Zoster Virus reactivation was mainly associated with BioNTech, and men were more affected than women. It has also been said that Herpesvirus 6 and 7 reactivation is associated with pityriasis rosea‐like eruption.^6^ Our cases of herpes simplex and pityriasis rosea‐like eruption occurred in male patients following the first dose of BioNTech, whereas one female patient suffered herpes zoster following the third dose of CoronaVac.

COVID‐19 vaccine hesitancy is a critical problem that hinders the adequate immunity of the population with vaccines. Informing physicians about vaccine‐related cutaneous reactions and reassuring and encouraging patients to take the next dose by suggesting the necessary medical treatments will contribute to the smoother progress of vaccination programs.

## INFORMED CONSENT

Written informed consent from the patients for the use of images and publication of their details were obtained.

## CONFLICT OF INTEREST

Both authors declared no conflict of interest.

## Data Availability

The data that support the findings of this study are available from the corresponding author upon reasonable request.
